# Teeth Baring as a Model to Understand Complex Facial Signals in a Tolerant Macaque Species

**DOI:** 10.1002/ajp.23697

**Published:** 2024-11-17

**Authors:** Federico Fantoni, Veronica Maglieri, Nicolò Giusti, Chiara Scopa, Virginia Pallante, Antonio Lorenzo Loprete, Elisabetta Palagi

**Affiliations:** ^1^ Department of Biology Unit of Ethology, University of Pisa Pisa Italy; ^2^ Department of Neurosciences University of Parma Parma Italy; ^3^ The Netherlands Institute for the Study of Crime and Law Enforcement Amsterdam The Netherlands

**Keywords:** agonistic context, *Macaca tonkeana*, peaceful context, playful context, signal specificity, signal stereotypy

## Abstract

Facial communication regulates many aspects of social life in human and nonhuman primates. Empirically identifying distinct facial expressions and their underlying functions can help illuminate the evolution of species' communicative complexity. We focused on bared‐teeth faces (BTFs), a highly versatile facial expression in the tolerant macaque *Macaca tonkeana*. By employing a diverse array of techniques (MaqFACS, unsupervised cluster analysis, Levenshtein distance, NetFACS), we quantitatively discriminated two distinct BTFs: bared‐teeth (BT) and open mouth bared‐teeth (OMBT), and evaluated their distribution across peaceful, playful, and agonistic contexts. Neither BT nor OMBT were context‐specific, although BT frequently occurred during peaceful interactions and with low levels of stereotypy. OMBT was highly stereotyped during play, a context involving strong unpredictability. The presence of tongue‐protrusion during OMBT was exclusive to peaceful contexts whereas the presence of glabella‐lowering during BT and OMBT was specific to agonistic contexts. Hence, BT and OMBT per se are not context‐specific, but their contextual relevance hinges on the inclusion of specific key elements. Moving forward, concurrent analyses of stereotypy and specificity should extend beyond our study to encompass other primate and non‐primate species, facilitating direct comparisons and revealing how communicative and social complexity coevolve.

## Summary

- Multi‐technique approaches are fundamental to explore facial complexity in primates.
- The concurrent analysis of stereotypy and specificity highlights the plasticity of a facial display.
- A single facial expression can acquire multiple meanings depending on the social context.

## Graphical abstract

Image summarizing the main results of the study.

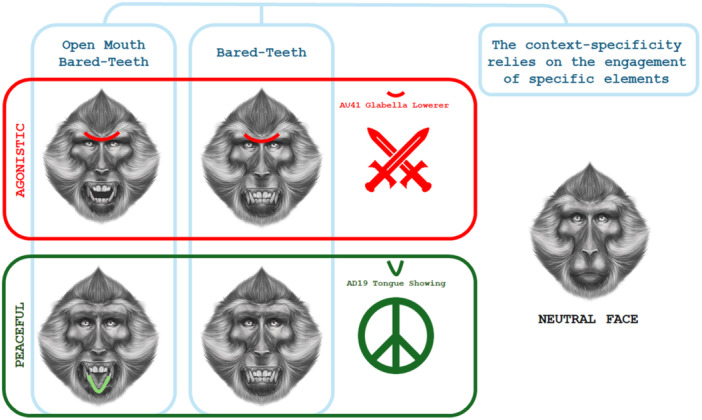

## 1 Introduction

A classical view of facial displays holds that they express the emotional states of the sender, with the communicative aspect arising as a by‐product deriving from the receiver experience (Darwin 1872; Buck 1994). Although the matching between facial expressions and emotions has been experimentally highlighted by several studies (Tomkins 1962; 1963; Levenson, Ekman, and Friesen 1990; Liu et al. 2017), focussing attention exclusively on the emotional aspect of facial displays introduces some methodological issues that can reduce the replicability of results (Waller, Whitehouse, and Micheletta 2017; Waller, Julle‐Daniere, and Micheletta 2020). One main issue is that facial displays are often defined on the basis of the supposed underlying emotion (Waller, Whitehouse, and Micheletta 2017). An elucidating example is the Silent Bared‐Teeth display (SBT), which has a negative emotional valence (e.g., submission, subordination) in most anthropoid species, while in some species like Sulawesi macaques (including Tonkean macaques, *Macaca tonkeana*), bonobos (*Pan paniscus*) and humans (*Homo sapiens*), it is mainly used to communicate affiliative intentions with a positive valence (van Hoof 1967; Thierry et al. 1989; Preuschoft 1992; Maestripieri 1997; Beisner and McCowan 2014; Vlaeyen et al. 2022). Because of such functional bivalence, SBT is often referenced with terms that indicate opposite affective states like fear (fear grimace) and pleasure (smile) depending on the species (Preuschoft and van Hooff 1995). Therefore, the use of emotions to reference facial expressions may affect the way researchers perceive them, potentially leading to confusing descriptions and ambiguity in the study of facial communication (Waller et al. 2008).

This issue can be solved by putting the spotlight on the morphology of facial expressions before exploring their functional nature (Waller, Julle‐Daniere, and Micheletta 2020). The morphological approach to the study of facial expressions allows detection of possible homologies because it focuses on the physical substrate producing the display (i.e., facial musculature), which is continuous across species and indicative of a common origin (van Hooff 1967; Preuschoft and van Hooff 1995; Waller, Whitehouse, and Micheletta 2008, 2017, 2020).

The Facial Action Coding System (FACS) is a valuable quantitative tool to describe facial expressions in humans (Human FACS, Ekman & Friesen 1978) and other species (e.g., MaqFACS, Parr et al. 2010). Via FACS it is possible to associate an unequivocal code to a specific morphological change in the face (AU). Therefore, FACS helps not only to quantify the complexity of a single facial expression objectively (e.g., number of AUs present in a given display, Waller, Caeiro, and Davila‐Ross 2015), but also the diversity and numerosity of the facial displays of certain species thus favouring objective comparisons (Parr et al. 2007; Rincon et al. 2023). Yet, determining the exact number of facial displays is not as easy as it sounds, because of the graded nature of facial expressions (Thierry et al. 1989; Clark et al. 2020; Clark et al. 2022).

In this study, we investigated the facial displays of Tonkean macaques (*Macaca tonkeana*), a species showing a high communicative complexity related to its highly tolerant social style (Thierry 2004, 2007; Freeberg, Dunbar, and Ord 2012; Rincon et al. 2023). We focused on “bared‐teeth faces” (BTFs) as a model category of facial expressions. According to the literature, BTFs include silent bared‐teeth, half‐open mouth, open‐mouth bared‐teeth, and open‐mouth bared‐teeth scream (van Hooff 1967; Thierry, Iwaniuk, and Pellis 1989, 2000; Pellis et al. 2011; Scopa and Palagi 2016; Table S1). However, previous categorization of BTFs was based mainly on their context or their combination with a vocalization and/or postures, and not on morphological facial output. In fact, these facial expressions resemble each other, especially regarding mouth and lip configuration thus introducing a certain level of ambiguity (Thierry et al. 1989; Clark et al. 2020).

Following the literature (van Hooff 1967; Thierry, Iwaniuk, and Pellis 1989, 2000; Pellis et al. 2011; Scopa and Palagi 2016), we hypothesized that there are two structurally different displays of BTF in Tonkean macaques: the bared‐teeth (BT), where the lips and mouth corners are retracted exposing teeth, and sometimes the gums, and the open‐mouth bared‐teeth (OMBT), where a wide opening of the jaw is added to the bared‐teeth configuration. In this case, we expected that BT and OMBT would differ in terms of which AUs they include (*Prediction 1*).

If the two facial configurations convey different meanings, we predicted that they would be expressed under different contexts, with BT more frequent during peaceful interactions than in other contexts (*Prediction 2a)* and OMBT relatively more frequent during interactions with competitive elements such as playful and agonistic contacts, as the literature suggests (Thierry, Iwaniuk, and Pellis 1989, 2000, Pellis et al. 2011; Scopa and Palagi 2016; Clark et al. 2020) (*Prediction 2b*).

FACS also facilitates the study of flexibility of a facial display, including how external factors can affect display complexity such as the stereotypy and specificity of the recruited AUs. For example, in crested macaques (*M. nigra*), a species closely related to Tonkean macaques, the application of MaqFACS (Parr et al. 2010) revealed that the SBT comprises four morphological variants related to the social context. Interestingly, SBTs displayed during submission, playful or sexual interactions were characterized by the presence of a specific AU in each context (Clark et al. 2020). SBTs during affiliative contacts were characterized by higher overall variability (Clark et al. 2020). Under particularly unpredictable situations such as during play fighting, the risk of misunderstanding can be high, and clearer signals should have been positively selected to reduce such risk (*risk reduction hypothesis*; Clark et al. 2022). By contrast, peaceful contexts may allow a higher degree of freedom in the performance of facial expressions. Therefore, we hypothesized an effect of context on the level of stereotypy (Margoliash, Staicer, and Inoue 1991; Maglieri et al. 2022, 2024) and specificity (Mielke et al. 2022) of the facial expressions in Tonkean macaques. Since we predicted that OMBTs would be used mainly in managing competitive interactions, we expected OMBTs to show a higher degree of stereotypy (*Prediction 3a*) and specificity (*Prediction 3b*) than BTs.

## 2 Methods

### 2.1 Ethical Note

This study was performed in full accordance with the American Society of Primatologist (ASP) Principles for the Ethical Treatment of Nonhuman Primates. The study was purely observational and did not involve manipulation of animals and no special permission was needed to perform the study. The researchers spent time getting the animals familiar to their presence. The data collection started when macaques did not show interest in the observers' presence. Signs of distress, fear, or aggression were never recorded towards the observers.

### 2.2 Subjects, Housing, and Field Data Collection

The study was conducted on 67 Tonkean macaques hosted at the Parc Zoologique de Thoiry (France). The data collection included 69 individuals in 2010 and 2011 and 60 individuals in 2014. Although most of the animals were individually recognized through facial and body features, we removed from the analyses those individuals that could not be reliably distinguished (see below). Kinship relations were unknown. The enclosure comprised an indoor area (182 m^2^) connected to an outdoor grassy area (3900 m^2^) enriched with pools, ropes, platforms, bushes, and trees. Table S2 shows the group composition over the years. Food was distributed twice a day at 12.00 a.m. and 06.00 p.m., and water was available ad libitum. In 2010 and 2011, C.S. carried out observations with the aid of a video camera (Samsung SMX‐F30BP, accuracy 0.02 s). In 2014, V.P. and a field assistant conducted the data collection using two video cameras simultaneously (JVC‐full‐HD‐GZE100SE and SONY‐DCR‐SR52, accuracy 0.02 s). We used sub‐group animal sampling (Altmann 1974). Given the tendency of macaques to aggregate in temporary and fluid sub‐groups, we could video‐record several subjects (up to 15 individuals) concurrently. We recorded a total of about 450 h of videos.

### 2.3 Video Analysis and Operational Definitions

We selected video footage where the faces of the subjects were clearly visible, yielding 40 h of video to analyse frame‐by‐frame by two coders using PotPlayer. We recorded all BT and open mouth bared teeth (OMBT) events (Table S1). In BT, the upper lip, or both lips are retracted, and the mouth corners are drawn back exposing the teeth and sometimes the gums, while the teeth are clenched or slightly separated. In OMBT, the upper lip, or both lips are retracted, and the mouth corners are drawn back exposing the teeth and sometimes the gums, while the mouth is widely open. For each BT and OMBT event, we noted the presence/absence of screaming, and assigned a specific social context (peaceful, playful, or aggressive). We based context assignment on the sender's behavior before and after facial display. Peaceful contexts included grooming, embracing, contact sitting, touching with hands or feet, and body sniffing. The playful context included play fighting (e.g., play wrestling, sparring, biting, jumping over). The agonistic context included not only aggressive behavior (e.g., charging/fleeing, grabbing, crouching, lunging, vocal and facial threats) but also acts of submission or appeasement that can end a social conflict. We tested interobserver reliability (Kaufman and Rosenthal 2009) for subject identity, BT and OMBT, and context as follows. About 10% of videos containing peaceful, play fighting and agonistic events were randomly extracted by the second author and assigned to the first and third authors, who analysed them independently. Cohen's kappa (Cohen 1960) was 0.85 for the individual recognition, 0.90 for BT, 0.93 for OMBT, 0.99 for peaceful context, 0.87 for playful context and 0.90 for agonistic context.

Using MaqFACS and its extensions (rhesus macaques: Parr et al. 2010; Barbary macaques: Julle‐Danière et al. 2015; crested macaques: Clark et al. 2020; Japanese macaques: Caeiro, Holmes, and Nishiwaki 2021), the BT events were analysed by the first and the third author who are MaqFACS official coders. FACS coding requires that the face and the onset and offset of the expression are clearly visible, so that one can evaluate facial changes resulting from muscle activation. For this reason, from a total of 1698 BTs (*N*_subjects_ = 62; mean 27.39 ± SD 22.20) and 1480 OMBTs (*N*_subjects_ = 64; 23.12 ± 25.68), we were able to analyse via MaqFACS 763 BTs (*N*_subjects_ = 61; 12.51 ± 11.40) and 466 OMBTs (*N*_subjects_ = 56; 8.32 ± 8.83) (Table S3 for details).

We started coding once the expression reached its apex, noting the absence/presence (0, 1) for each action unit (AU). Because we aimed to quantitatively evaluate the diversity of bared‐teeth expressions, and not their combinability with other expressions, we discarded the Action Descriptor AD101 (scalp retraction) and EAU3 (ear flattener), as these movements are associated with a distinct well‐documented facial expression, *scalp retraction*, which can occur either alone or in combination with other facial expressions (Thierry, Iwaniuk, and Pellis 2000). We never recorded AU25 without AU26 or AU27. We did not take into account the EAUs because of the dark fur covering the head of the animals that makes such EAUs not always clearly visible. We calculated interobserver reliability for 131 display observations (10.7%) using the following formula (Ekman and Friesen 2002):

$$\mathrm{Agreement}=\frac{2(\mathrm{number of AUs agreed by both coders})}{\left(\mathrm{AUs coded by coder}\;1\right)+(\mathrm{AUs coded by coder}\;2)}.$$

The agreement coefficient obtained between the two coders was 0.92.

### 2.4 Data Analysis

#### 2.4.1 Two Different Bared‐Teeth Displays

To test our a priori classification of the BTFs based on the AUs included we ran a principal component analysis (PCA) of mixed data (“PCAmixdata” package in R; Chavent et al. 2014), which uses a generalized singular value decomposition (GSVD) of processed data. This approach includes the PCA, and multiple correspondence analysis (MCA) as special cases, allowing the integration of categorical variables (Chavent et al. 2014). We removed from the analysis all AUs that were observed in less ≤ 1% of cases. Then we performed a *k‐means unsupervised clustering* (*k* = *2*) by one‐hot encoding the data to group the observations and visualize the data (Hartigan and Wong 1979). We created a dummy variable for each value in each category, then transformed the categorical variables into a one‐hot vector representation. To avoid bias related to the over‐representation of certain individuals in the data, we randomly excluded 169 events from those individuals in the upper quartile of the distribution. Moreover, we also removed events performed by juveniles in 2014, as their identity was unknown. The final number of BTFs was 1047. We used Fisher exact tests to see which AUs differed between BT and OMBT. See Supplementary Material.

#### 2.4.2 The Emission of Different BTFs is Affected by the Context

To test if performance of BTFs was affected by social context, we ran a Generalized Linear Mixed Model (GLMM; glmmTBM package in R; Brooks et al. 2017; R Core Team, 2020; version 1.4.1717) setting the facial expression (BT or OMBT) as response variable (binomial error distribution; *N*_cases_ = 1216). Fixed factors were *sex* (male/female), *age* (adult/subadult/juvenile), *social context* of occurrence (peaceful/playful/agonistic) and *screaming* (presence/absence), while sender's identity was a random factor. Because the sex of juveniles was unknown in 2014, we excluded them from the model. Fixed factors showed low collinearity (VIF_min_ = 1.01; VIF_max_ = 1.61). We compared this model to a control model including the random factor and the fixed factor *screaming*, using the “*ANOVA"* function in R. Finally, to perform pairwise comparisons of predictor variables, we used estimated marginal means with Tukey adjustment via the ‘*emmeans*’ package in R (Lenth 2023). See Supplementary Material.

#### 2.4.3 Degree of Stereotypy

We used the Levenshtein Distance (LD) to measure stereotypy of BT and OMBT among individuals (Margoliash, Staicer, and Inoue 1991; Maglieri et al. 2022, 2024). LD assesses the difference between two data sequences, here a series of AUs, by calculating the minimum number of changes (i.e., insertions, deletions, substitutions) needed to transform one sequence into another; accordingly, a lower LD represents greater similarity (Kohonen 1985). To perform this analysis, we limited our data set to those individuals whose identity was known and for whom we had coded at least two BTs and two OMBTs in each context. We transformed each AU into an alphabetical letter (e.g., AU1 + 2 → A) and thus each facial expression was represented by a string of letters. We calculated LD separately for BTs and OMBTs using the “*stringdistmatrix”* function (stringdist R package; Loo 2014). This procedure generated six square matrices (BTs and OMBTs in each of the three contexts) representing ‘the distances’ between pairs of string. We subsequently combined the matrices into a single one by maintaining the information about the facial expression performed (BTs and OMBTs) and the context. To test if the stereotypy degree of the two expressions varied across the different contexts in each context, we ran a Linear Mixed Model (LMM, glmmTBM package in R; Brooks et al. 2017) setting the LD value as response variable (Gaussian error distribution; *N*_cases_ = 118500). The fixed factor was the interaction between the *context* and the *facial expression* with the factors showing low collinearity (VIF_MIN_ = 1.37; VIF_MAX_ = 1.37). The interaction between the senders’ identities was set as random factor. We compared the full model to a null model including only the random factor. See Supplementary Material.

#### 2.4.4 Context Specificity

To calculate context specificity (i.e., the conditional probability of an AU or an AUs' combination being associated with a context), we used the function *‘specificity’* in the R package “NetFACS” (version 0.5.0; Mielke et al. 2022). Specificity is a ratio expressing the number of times a combination was observed in a given context divided by the number of times it was observed across all contexts (Rincon et al. 2023). It ranges from 0 (an AU combination never occurred in a given context) to 1 (it occurred exclusively in this context). We calculated specificity values, separately for BTs and OMBTs, for all AU combination sizes (ranging from 1 to 7). We then ran an LMM, setting *specificity* as the response variable (Gaussian error distribution, *N*_cases_ = 858), and the context*facial expression as fixed factor. The AU combination size was set as random factor to control for the reiteration of each AU. The fixed factors showed low collinearity (VIF_MIN_ = 1.02; VIF_MAX_ = 1.02). We compared the full to a null model comprising only the random factor. See Supplementary Material.

## 3 Results

### 3.1 Two Different Bared‐Teeth Displays (Prediction 1 Supported)

**Figure 1:**
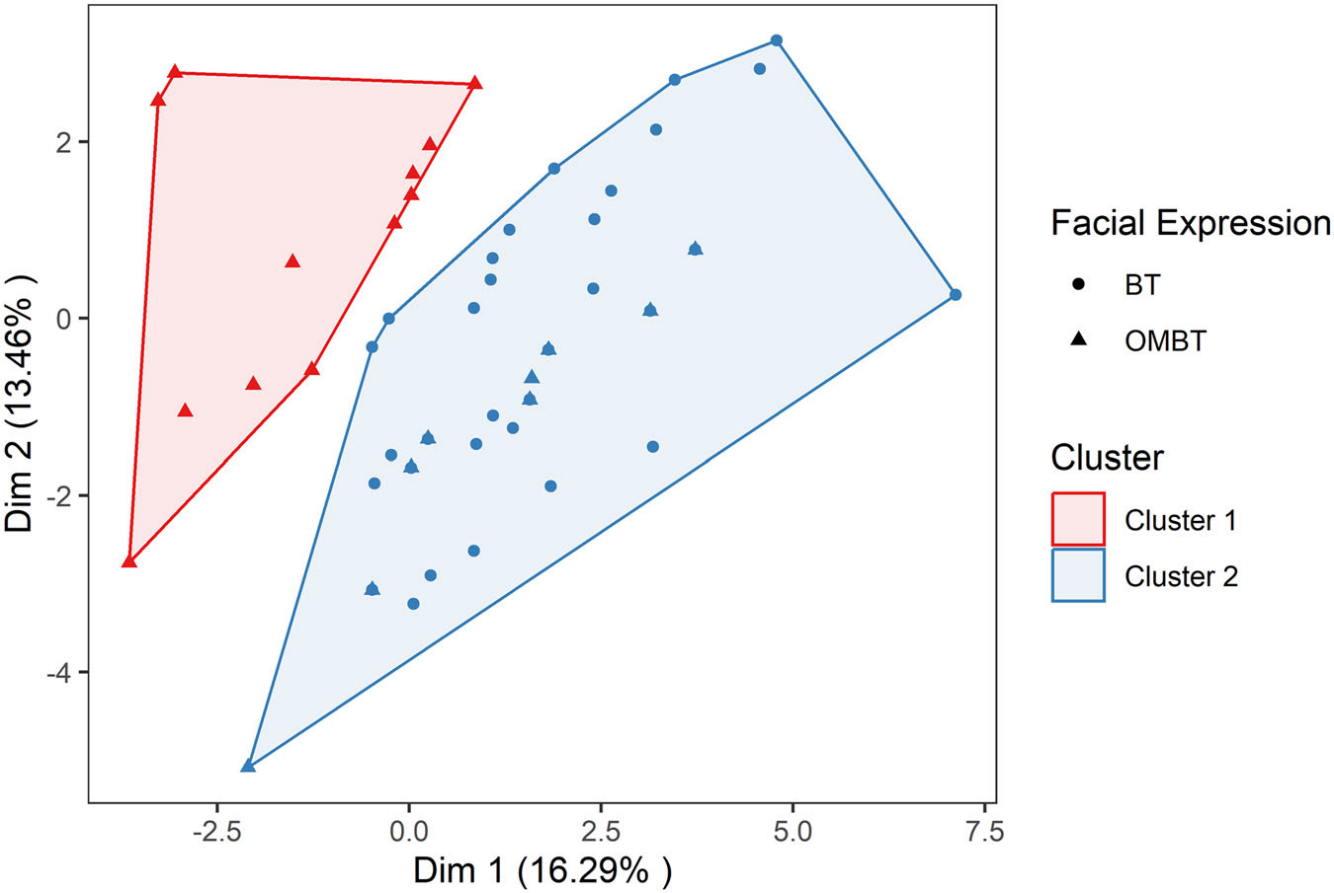
Bi‐dimensional representation of the sample of facial expressions obtained by initializing the principal component analysis of mixed data (*PCA*mix) combined with the k‐means clustering results. The differently shaped points on the map (circles and triangles) reflect the a priori classification of Bared‐Teeth (BT) and Open‐Mouth Bared‐Teeth (OMBT); the different colored clouds represent the distribution of BT and OMBT identified by the k‐means clustering.

The a priori classification of BT and OMBT was confirmed by the analyses. The first cluster identified by the k‐means included 88.5% of OMBTs, while the second cluster included all BTs and 11.5% of OMBTs (Figure 1). The results of the exact Fisher test and the power test are reported in Table 2. All subsequent analyses of BT and OMBT are based on the outcome of this k‐means unsupervised cluster analysis.

### 3.2 The Emission of Different BTFs is Affected by the Context (Prediction 2a Supported and 2b Not Supported)

**Figure 2:**
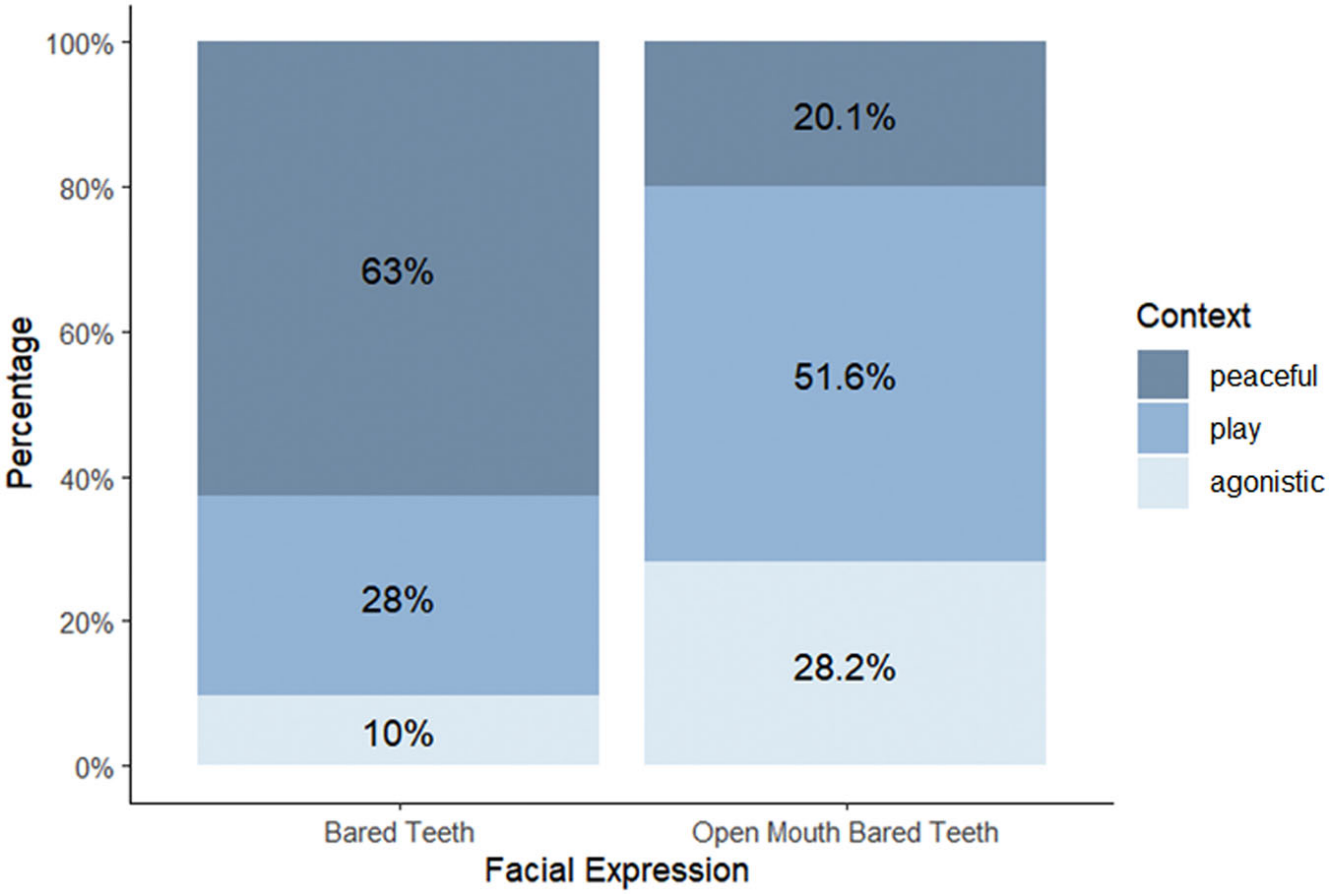
Percentages of bared‐teeth (BT) and open mouth bared‐teeth (OMBT) in relation to social context (affiliation = dark blue; play = medium blue; aggression = light blue).

The full model differed from the control model (*χ* ^2^ = 1670.94, df = 4, *p* < 0.001, *N*_cases_ = 1216). *Context* had a significant effect on the type of BTF (Table 1A). The probability of BT emission was higher during peaceful than playful (post‐hoc Tukey pairwise *t*‐ratio_peaceful versus playful_ = −11.269, df = inf, *p* < 0.001) and agonistic contexts (*t*‐ratio_peaceful versus agonistic_ = −7.294, df = inf, *p* < 0.001). The probability of BT and OMBT did not differ between playful and agonistic contexts (*t*‐ratio_playful versus agonistic_ = 0.397, df = inf, *p* = 0.917) (Table 1; Figure 2).

**Table 1:** Estimated parameters (Coeff), Standard Error (SE), and results of the Likelihood Ratio Tests (χ^2^) of the models. Significant *p* values are shown in bold.

***A ‐ Prediction 2 (GLMM) – The emission of different bared‐teeth displays is affected by the immediate social context (partially supported) – Response Variable: BT/OMBT***					
**Fixed effects**	**Coeff**	**SE**	**χ** ^**2**^	**df**	***p***
Intercept	−1.738	0.204	n/a	n/a	n/a
**Tested variables**					
Sex	−0.327	0.249	1.723	1	0.189
Age	0.535	0.946	0.320	1	0.572
Context			141.711	2	**< 0.001**
Context_[PLAY]_	1.970	0.174			
Context_[AGONISTIC]_	1.866	0.255			
**Control variable**					
Screaming	1.326	0.305	18.933	1	< 0.001
*N*_cases_ = 1216, *N*_ID_ = 63, Variance for the random factor: ID = 0.440 ± 0.663 SD					

***B ‐ Prediction 3a (LMM) – OMBTs show a higher degree of stereotypy than BTs, especially in agonistic and playful context (supported) ‐ Response Variable: LD values***					
**Fixed effects**	**Coeff**	**SE**	**χ** ^**2**^	**df**	***p***
Intercept	−1.606	0.499	n/a	n/a	*n/a*
Context			675.32	2	**< 0.001**
Context_[PLAY]_	−0.015	0.015			
Context_[AGONISTIC]_	−0.468	0.030			
Facial expression	−0.626	0.024	4093.31	1	**< 0.001**
Context*Facial expression			151.19	2	**< 0.001**
Context_[PLAY]_*Facial expression	−0.606	0.027			
Context_[AGONISTIC]_*Facial expression	0.355	0.040			
*N*_cases_ = 118500, *N*_ID1_ = 58, *N*_ID2_ = 58, Variance for the random factors: ID1 = 0.051 ± 0.226 SD; ID2 = 0.091 ± 0.301 SD					

***C ‐ Prediction 3b (LMM) – OMBTs show a higher degree of specificity than BTs, especially in agonistic and playful context (not supported) ‐ Response Variable: Specificity values***					
**Fixed Effects**	**Coeff**	**SE**	**χ** ^**2**^	**df**	***p***
Intercept	0.445	0.031	n/a	n/a	*n/a*
Context			31.782	2	**< 0.001**
Context_[PLAY]_	−0.065	0.032			
Context_[AGONISTIC]_	−0.041	0.032			
Facial expression	0.0125	0.029	60.042	1	**< 0.001**
Context*Facial expression			16.592	2	**< 0.001**
Context_[PLAY]_*Facial expression	−0.104	0.045			
Context_[AGONISTIC]_*Facial expression	0.080	0.041			
*N*_cases_ = 858, *N*_COMBINATION SIZE_ = 7, Variance for the random factor: COMBINATION SIZE = 0.003 ± 0.050 SD; ID2 = 0.091 ± 0.301 SD					

Abbreviations: BT, bared teeth; df, degree of freedom; LD, Levenshtein distance; n/a, not applicable; OMBT, open mouth bared teeth.

### 3.3 OMBTs are More Stereotyped Than BTs Especially Under Potentially Risky Situations (Prediction 3a Supported)

**Figure 3:**
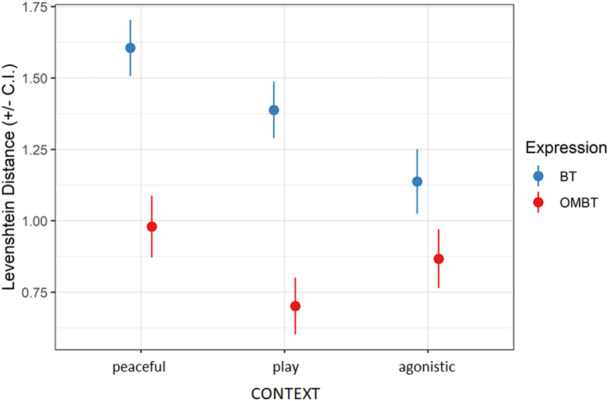
Degree of stereotypy of bared‐teeth (BT, blue) and open mouth bared‐teeth (OMBT, red) measured via the Levenshtein Distance across the three different social contexts considered in the study.

The full model of LD differed significantly from the null model (χ^2^ = 8473.9, df = 5, *p* < 0.001, *N*_cases_ = 118500). *Facial expression*, *context* and their interaction were significant predictors of the facial movement (Table 1B). Overall, BTs were less stereotyped than OMBTs in each context (peaceful, *t*‐ratio = 25.647, df = 118491, *p* < 0.001; play, *t*‐ratio = 59.720, df = 118,491, *p* < 0.001; agonistic, *t*‐ratio = 8.459, df = 118491, *p* < 0.001; Figure 3).

BTs in peaceful contexts were less stereotyped than in playful and agonistic contexts (*t*‐ratio_peaceful versus playful_ = 22.013, df = 118491, *p* < 0.0001; *t*‐ratio_peaceful versus agonistic_ = 15.697, df = 118491, *p* < 0.0001). BTs in a playful context were less stereotyped than those in an agonistic context (*t*‐ratio_playful versus agonistic_ = 8.134, df = 118491, *p* < 0.0001; Figure 3).

OMBTs in peaceful context were also less stereotyped than those in playful and agonistic contexts (*t*‐ratio_peaceful versus playful_ = 10.679, df = 118491, *p* < 0.0001; *t*‐ratio_peaceful versus agonistic_ = 3.724, df = 118491, *p* = 0.0027). OMBTs in an agonistic context were less stereotyped than in a playful context (*t*‐ratio_playful versus agonistic_ = −8.613, df = 118491, *p* < 0.0001; Figure 3).

### 3.4 OMBT Specificity is Higher Than BT Specificity Especially Under Risky Situations (Prediction 3b Not Supported)

**Figure 4:**
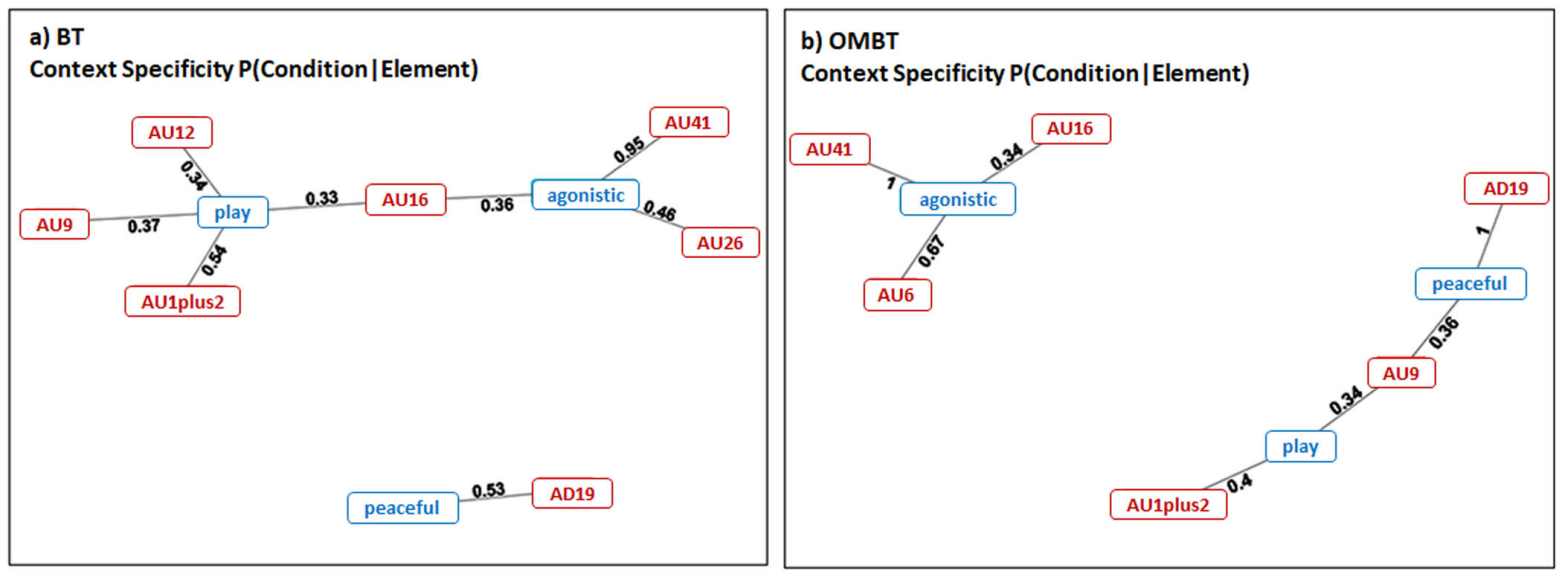
Network representing the probability of an AU being present in each context during a BT (i.e., the concurrent context specificity) (a) and OMBT (b). Red squares represent the AU and blue squares represent the contexts. Only the edges scoring a *p* < 0.01 are visualized.

**Figure 5:**
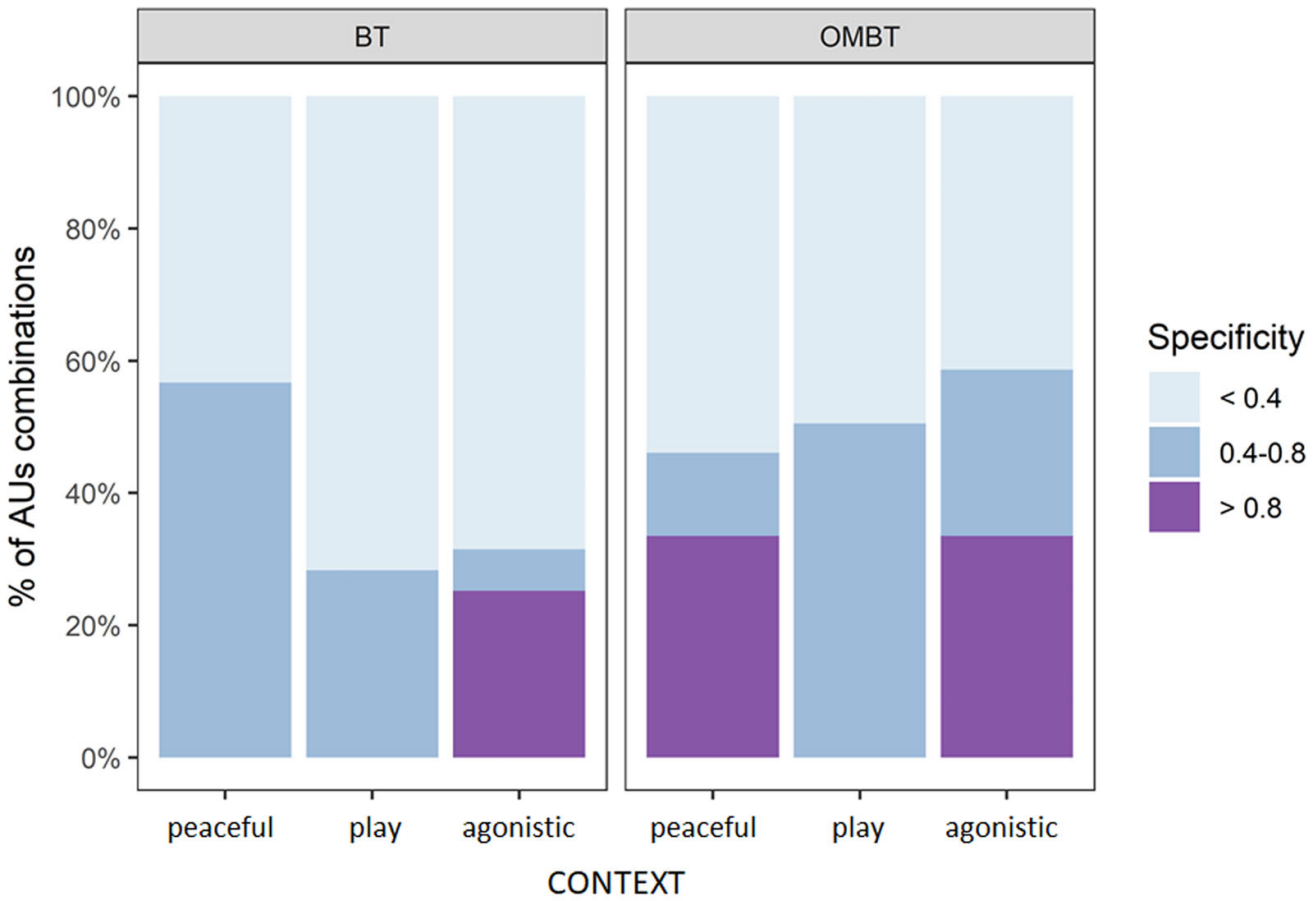
Percentages of AUs combination emitted with high ( > 0.8), medium (0.4–0.8), and low ( < 0.4) specificity for BT (left) and OMBT (right). Specificity ranges from 0 to 1. 0 indicates that an AU was never observed in a given context, 1 indicates that an AU was uniquely observed in that context.

The networks representing the probability of each context to be present when an AU was active (specificity) for BT and OMBT are visualized in Figure 4a,b respectively. Among BTs, the AU combinations with high specificity (> 0.8) were found only in the agonistic context (25%; Figure 5). In playful and peaceful context, no combinations had high specificity values (Figure 5). For OMBT, the AU combinations with high specificity (> 0.8) were found in the agonistic and peaceful context (34% for both). In the playful context, no combinations scored high specificity values (Figure 5).

**Figure 6:**
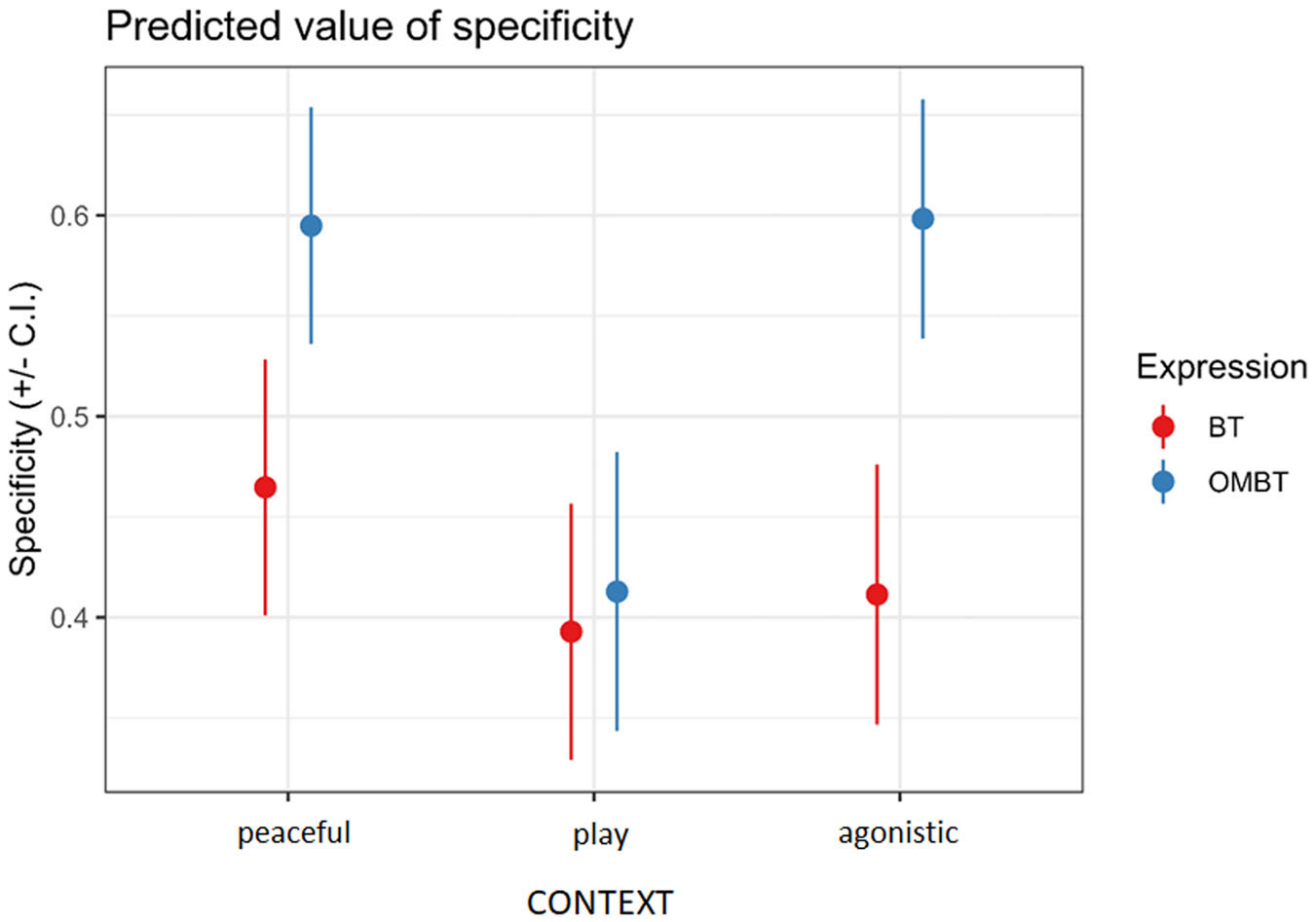
Degree of stereotypy of bared‐teeth (BT, blue) and open mouth bared‐teeth (OMBT, red) measured via the Levenshtein Distance across the three different social contexts considered in the study.

The full model differed significantly from the null model (χ^2^ = 105.14; df = 5, *p* < 0.001, *N*_cases_ = 858). The variables *context*, *facial expression* and their interaction affected the AU combination specificity (Table 1, Figure 6). Within BTs, specificity did not differ across contexts (*t*‐ratio_peaceful versus playful_ = 2.306, df = 850, *p* = 0.193; *t*‐ratio_peaceful versus agonistic_ = 1.708, df = 850, *p* = 0.527; *t*‐ratio_agonistic versus playful_ = 0.592; df = 850; *p* = 0.992; Table 1). Within OMBTs, specificity of the playful context was significantly lower than peaceful (*t*‐ratio_peaceful versus playful_ = 5.823, df = 850, *p* < 0.001) and agonistic context (*t*‐ratio_agonistic versus playful_ = 5.947, df = 850, *p* < 0.001). OMBT specificity did not differ between peaceful and agonistic contexts (t‐ratio_peaceful versus agonistic_ = −0.135; df = 850; *p* = 1.000).

BT scored a lower specificity than OMBT under peaceful (*t*‐ratio_peacefulBT versus peacefulOMBT_ = −5.216, df = 850, *p* < 0.001) and agonistic context (*t*‐ratio_agonisticBT versus agonisticOMBT_ = −7.576, df = 850, *p* < 0.001). The specificity of BT and OMBT did not differ in the playful context (*t*‐ratio_playfulBT versus playfulOMBT_ = −0.478, df = 850, *p* = 0.997) (Table 1, Figure 6).

## 4 Discussion

Using Tonkean macaque facial expressions as a study system, we revealed a standardized distinction between two similar expressions (bared‐teeth and open‐mouth bared‐teeth) illustrating an iterative approach that can be applied to other facial expressions. Initially, BT and OMBT were distinguished a priori based on existing literature (Table S1). Subsequently, using MaqFACS (Parr et al. 2010) we coded specific AUs for each facial expression and subjected the AUs to a rigorous clustering procedure using an unsupervised k‐means algorithm. The resulting clusters aligned with the a priori classification of the two bared‐teeth facial expressions in Tonkean macaques (*Prediction 1* supported, Figure 1), which differed mainly in the degree of mouth‐opening (BT, AU25 or AU25 + 26; OMBT, AU25 + 27; Table 2).

**Table 2:** Comparison of the action units (AUs) and action descriptor (AD) observed in BT and OMBT, the relative *p* values obtained by the Fisher's exact test (significant *p* values in bold) and the results of the power test. The columns BT and OMBT report the exact number of times AUs/AD is included in the facial expression. (%) indicates the percentages of occurrence of specific AUs/AD in BT and OMBT.

	BT (%)	OMBT (%)	Fisher	Power test
AU1 + 2	86 (12.9%)	60 (15.3%)	0.269	0.23
AU41	4 (0.6%)	7 (1.8%)	0.111	0.41
AU6	0 (0%)	7 (1.8%)	**< 0.001**	0.92
AU43	0 (0%)	4 (1.0%)	0.02	0.58
AU9	491 (73.5%)	363 (92.6%)	**< 0.001**	1
AU10	667 (99.9%)	392 (100%)	1.000	0
AU12	635 (95.1%)	389 (99.2%)	**< 0.001**	0.99
AU16	571 (85.5%)	379 (96.7%)	**< 0.001**	1
AU26	284 (42.5%)	45 (11.5%)	**< 0.001**	1
AU27	0 (0%)	347 (88.5%)	**< 0.001**	1
AD19	47 (7.0%)	3 (0.007%)	**< 0.001**	1

The morphological separation of BT and OMBT did not completely reflect their context specificity. Neither expression was exclusively performed during a specific context, although BT was mostly displayed in peaceful contexts, and was probably associated with a relaxed affective state (*Prediction 2a* supported). We noted that BT and OMBT are occasionally used as a submissive signal by one partner, but are more often used for reconciliation (Thierry et al. 1989). In Tonkean macaques, affiliative facial displays (e.g., lipsmacking, BT, OMBT) that end a conflict are often accompanied by conciliatory contacts. Contrary to expectations, BT and OMBT performance did not differ between playful and agonistic contexts, indicating some flexibility in the use of these facial expressions during risky situations (*Prediction 2b* not supported). The absence of a preferential emission of one of the two facial expressions during an agonistic event could relate to the sender's role as aggressor or victim. Because we did not consider whether the facial expression was emitted by the attacker or the victim, it is possible that the coarse grouping under the “agonistic” context may have concealed differences in signaling. We therefore recommend that future studies focus on the direction of the agonistic behavior to clarify the use of these signals in agonistic contexts.

When Tonkean macaques are playing, OMBT substitutes for the *relaxed open mouth* (ROM) display, a metacommunicative playful signal that is widespread among primates (van Hoof 1967; Thierry et al. 1989; Preuschoft and van Hooff 1995; Pellis et al. 2011; Palagi, Norscia, and Spada 2014). In several primates, ROM involves wide opening of the jaw, but without retraction of the mouth corners (Preuschoft 2000). The substitution of ROM with OMBT in Sulawesi macaques may be linked to the motivational shift of the BT, from a submissive towards a peaceful meaning. In fact, evolutionarily speaking, the OMBT may reflect a merger of the BT and the *play face* (Preuschoft 2000), resulting in an expression that resembles the *full play face* well described in hominoids (Palagi 2008; Davila‐Ross, Owren, and Zimmermann 2009; Waller and Cherry 2012; Waller, Caeiro, and Davila‐Ross 2015; Bresciani, Cordoni, and Palagi 2022) and geladas (*Theropithecus gelada*; Palagi and Mancini 2011). The presence of BT and OMBT during play reflects the dual nature of this behavior, which can be both cooperative and competitive at the same time (Pellis and Pellis 1998; Reinhart et al. 2010; Palagi et al. 2016; Burghardt et al. 2024). Accordingly, we could hypothesize that as the degree of competition during play increases (i.e., more asymmetrical play session), the proportion of OMBTs compared to BTs should increase as well. However, another explanation might involve the arousal state of the interacting animals. For example, we could hypothesize that a higher arousal state in the sender (i.e., more intense play session) would lead to a higher proportion of OMBTs (Waller, Caeiro, and Davila‐Ross 2015). This idea is worthy of further investigation.

Our results indicate that BT and OMBT differ not only in the AUs they contain but also in their degree of stereotypy and the specificity of AU combinations. In particular, BT showed lower stereotypy and specificity than OMBT (*Prediction 3a* supported, Figure 3 and 5). These results support the *risk reduction hypothesis* as BTs were displayed mostly during peaceful contexts (Figure 2). In terms of BT stereotypy in each context, we found that the peaceful context predicted more variability than the agonistic and playful contexts (Figure 3). During affiliative interactions there is a weaker selective pressure to develop highly stereotyped facial expression that unequivocally manifest senders’ intentions, because misunderstandings are likely to have negligible consequences. This same pattern seems to hold for OMBT, which showed the highest Levenshtein Distance (LD) values during peaceful interactions (Figure 3). Surprisingly, OMBT scored the lowest LD values during play (Figure 3), a context requiring clear signals of motivation by both players to prolong the session (Mancini, Ferrari, and Palagi 2013; Scopa and Palagi 2016). Although comparisons with other primates are not possible at present, some comparisons can be made with social carnivores. A similar result was found in dogs whose playful facial expression (Maglieri et al. 2022), the ROM, is emitted in a highly stereotyped way showing LD values overlapping with those recorded for OMBT in Tonkean macaques. In dogs, the stereotyped ROM serves a metacommunicative function in maintaining the play session balanced between the players (Maglieri et al. 2022). A similar function may apply to Tonkean macaques, a species that engages in play at every age (Ciani et al. 2012).

Given the weak intensity and the high bidirectionality characterizing the aggressive interactions of Tonkean macaques (Ciani et al. 2012; Thierry et al. 2004), it is not surprising that OMBTs emitted during agonistic encounters can be more plastic and less stereotyped, probably because of the necessity of social modulation of the signal in tolerant species (Rincon et al. 2023). This result goes in tandem with the high level of AU specificity recorded for OMBT in both peaceful and agonistic contexts compared to the play context (*Prediction 3b* not supported, Figure 6). This finding could relate to the presence of specific AUs (not present during play) that can be recruited for unambiguous signaling for peaceful or agonistic purposes (Figure 5). Specifically, the network analysis (NetFACS, Mielke et al. 2022) revealed the presence of some context specific AUs (Figure 4). The AD19 (*tongue showing*) is present in the 100% of the OMBTs during peaceful contacts. The AU41 (*glabella lowerer*) occurs in the 100% of the OMBTs (Figure 4b) and in the 95% of BTs during agonistic encounters (Figure 4a). Accordingly, AU41 predicts the agonistic context in Tonkean macaques. A similar result has been found by Clark et al. (2022) in *Macaca nigra*, another tolerant macaque. The authors found that some AUs and Action Descriptors (ADs) distinguish social contexts such as play (*jaw stretching*, AU27), sex (*jaw wobble*, AD184), and submission (*teeth chattering*, AD182). In wolves, a socially complex carnivore, Maglieri et al. (2024) demonstrated that, despite sharing several AUs, the *full‐ROM* (analogous to the full play face in great apes) and the *high Threatening‐Face* differed in two highly context specific AUs. The brow lowerer (AU104) and gum exposure (GUM) were never present during play fighting and recruited only during real fighting. This also aligns with findings from studies of both humans (Waller et al. 2008; Shariff and Tracy 2011) and mice (Defensor et al. 2012), indicating that the narrowing of the eyelid opening is associated with increased aggression and may function as an indicator of dominance or an imminent threat.

Overall, our results show the multifaceted facial expression system of Tonkean macaques, with a single facial expression that can acquire multiple meanings depending on the social context. The use of BTs and OMBTs seems to be extremely flexible, reflecting the social complexity of the species (Rebout et al. 2020, 2021; Waller et al. 2022). Concurrent analysis of stereotypy and specificity would be valuable, as they would allow direct comparisons and could reveal the role of variability in signal emission as related to different degrees of social complexity and tolerance.

Our study has some limitations that need to be addressed in future work. First, vocalizations should be included in the analysis, because unimodal communication is generally rare in animals, whose signals often comprise elements from different sensory channels (Partan and Marler 1999; Micheletta et al. 2013). Other communicative elements can be associated with facial expressions (e.g., postures, gestures), determining different meanings. Finally, the identity of the receiver and its attentional state are crucial factors to be considered (*message‐meaning analysis*), since they can make the difference in the signal production, in shaping its designed features and clarifying precise functions.

## Author Contributions

Conceptualization: Elisabetta Palagi. Data curation: Federico Fantoni, Veronica Maglieri, Chiara Scopa, Virginia Pallante. Investigation: Federico Fantoni, Veronica Maglieri, Nicolò Giusti, Chiara Scopa, Virginia Pallante. Formal analysis: Federico Fantoni, Veronica Maglieri, Antonio Lorenzo Loprete, Elisabetta Palagi. Methodology: Federico Fantoni, Veronica Maglieri, Antonio Lorenzo Loprete. Supervision: Elisabetta Palagi. Validation: Elisabetta Palagi: Visualization: Federico Fantoni, Veronica Maglieri, Antonio Lorenzo Loprete. Writing–original draft: Federico Fantoni, Veronica Maglieri, Elisabetta Palagi. Writing–review and editing of the first version of the manuscript: Federico Fantoni, Veronica Maglieri, Nicolò Giusti, Chiara Scopa, Virginia Pallante, Antonio Lorenzo Loprete, Elisabetta Palagi.

## Conflicts of Interest

No competing interest to declare.

## Supporting information

Supporting information.

Supporting information.

Supporting information.

Supporting information.

Supporting information.

Supporting information.

Supporting information.

## Data Availability

All the raw data are submitted as Supplementary Material.
