# Supplementary material for: Teeth Baring as a Model to Understand Complex Facial Signals in a Tolerant Macaque Species
Source: Am J Primatol. 2024 Nov 17;87(1):e23697. doi: 10.1002/ajp.23697 (PMC11650955; doi:10.1002/ajp.23697)

**Table S1** - Descriptions of the bared-teeth facial expressions reported in literature for Tonkean macaques. The images are extracted from the videos analysed for the present study.


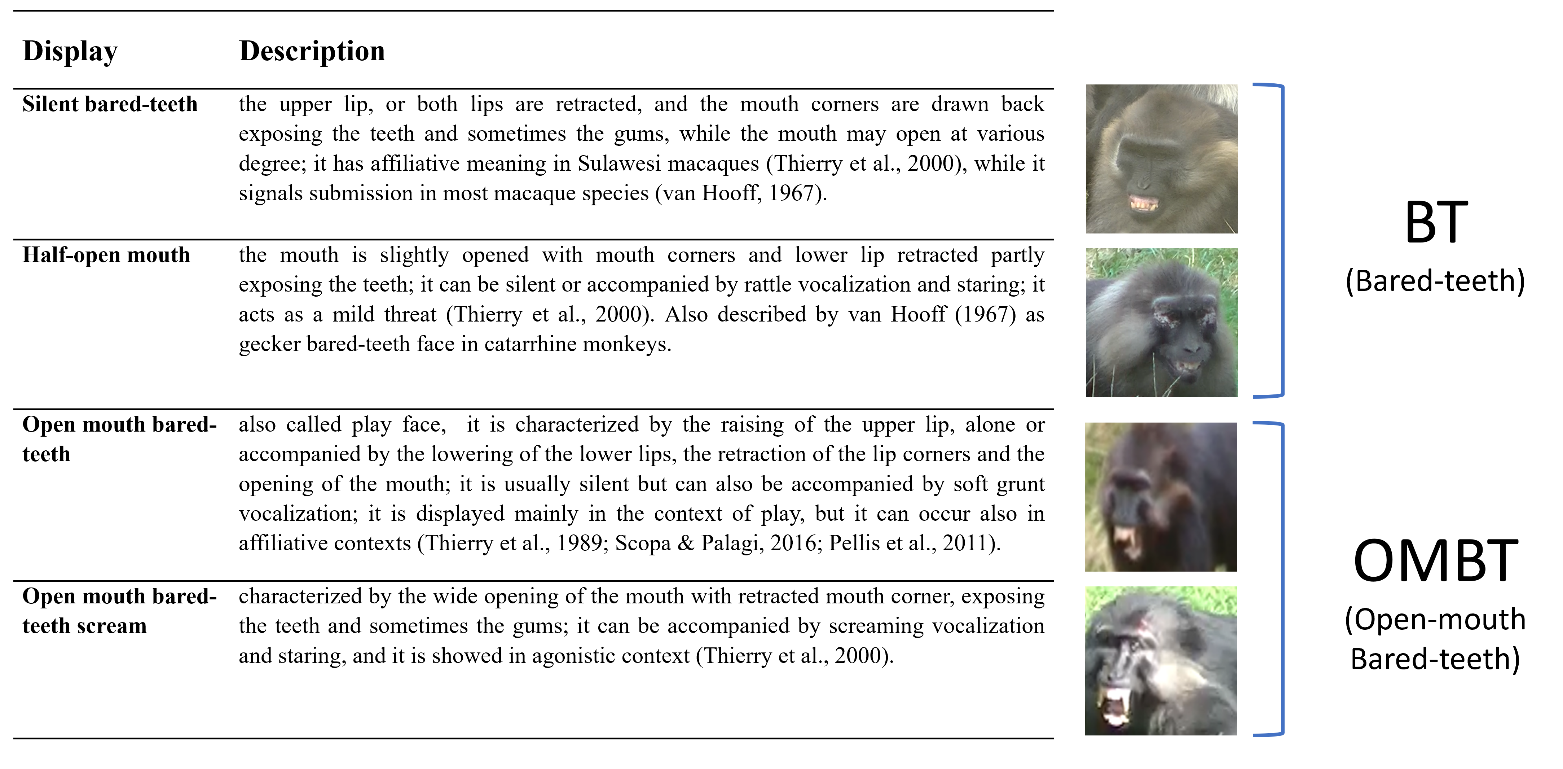

Supplement: Supplementary file 1 — Supporting information. [file AJP-87-e23697-s006.docx]
