# Supplementary material for: Teeth Baring as a Model to Understand Complex Facial Signals in a Tolerant Macaque Species
Source: Am J Primatol. 2024 Nov 17;87(1):e23697. doi: 10.1002/ajp.23697 (PMC11650955; doi:10.1002/ajp.23697)
Supplement: Supplementary file 2 — Supporting information. [file AJP-87-e23697-s004.docx]

| Group composition | | | | | | | |
| --- | --- | --- | --- | --- | --- | --- | --- |
| Subjects | **Sex** | **Age class 2010-11** | **Age class 2014** | **Subjects** | **Sex** | **Age class 2010-11** | **Age class 2014** |
| BR  CG  PG  TIR  VE  OU  SO  PT  GF  JK  NO  JG  KU  BF  PH  MRB  CD  PP  OT  PAP  MH  SU  SA  DM  LO  PR  PIN  PPU  AR  VS  SM  PA  CY  MO  DE (2011) | M  M  M  M  M  M  M  M  M  M  M  M  M  M  M  M  M  M  M  M  M  M  M  M  M  M  M  M  M  M  M  M  M  M  M | Ad  Ad  Ad  Ad  Ad  Ad  Ad  Ad  Ad  Ad  Subad  Ad  Ad  Ad  Ad  Ad  Ad  Ad  Ad  Ad  Ad  Ad  Ad  Ad  Ad  Ad  Ad  Ad  Ad  Ad  /  /  Juv  Juv  Juv | Ad  Ad  Ad  Ad  Ad  Ad  Ad  Ad  Ad  Ad  Ad  Ad  Ad  Ad  Ad  †  Ad  Ad  †  †  Ad  Ad  Ad  Ad  Ad  Ad  Ad  Ad  Ad  Ad  Subad  Subad  /  Subad  / | **AM**  **TR**  **PU**  **SY**  **AMB**  **CN**  **CP**  **ES**  **PC**  **GL**  **SDF**  **SI**  **NDT**  **SC**  **CO**  **OR**  **SBB**  **MG**  **BB**  **PI**  **PY**  **MB**  **RC**  **SB**  **CS**  **VO**  **DF**  **RP**  **MA**  **BT**  **GR**  **3N**  **3C**  PIC  PE  **IN**  **PAN**  **FR (2011)** | F  F  F  F  F  F  F  F  F  F  F  F  F  F  F  F  F  F  F  F  F  F  F  F  F  F  F  F  F  F  F  F  F  F  F  F  F  F | Ad  Ad  Ad  Subad  Ad  Ad  Ad  Ad  Subad  Ad  Ad  Ad  Ad  Ad  Ad  Ad  Ad  Ad  Ad  Ad  Ad  Ad  Ad  Ad  Ad  Ad  Ad  Ad  Ad  Ad  Ad  Juv  Juv  /  /  Juv  Juv  Juv | †  Ad  Ad  Ad  Ad  Ad  Ad  Ad  Ad  †  Ad  Ad  Ad  Ad  Ad  Ad  Ad  †  Ad  Ad  †  Ad  Ad  Ad  Ad  Ad  Ad  Ad  Ad  Ad  Ad  Ad  Ad  Ad  Ad  /  /  † |

**Table S2** Group composition across 2010, 2011, and 2014. For the age definition and categorization, we followed Liao et al. (2015). "PE" and "PIC" from 2014 correspond to "IN" and "PAN" from 2010/11, but it was impossible to establish who was whom. The same is for "SM" and "PA" respect to "CY" and "DE". “†” symbols indicate that the subject died or was transferred between 2011 and 2014.
