# Supplementary material for: Teeth Baring as a Model to Understand Complex Facial Signals in a Tolerant Macaque Species
Source: Am J Primatol. 2024 Nov 17;87(1):e23697. doi: 10.1002/ajp.23697 (PMC11650955; doi:10.1002/ajp.23697)
Supplement: Supplementary file 3 — Supporting information. [file AJP-87-e23697-s003.docx]

**Table S3** - Resuming distribution of the FACS-analysed BTs and OMBTs among social contexts and sexes; the unknown category refers to juveniles in 2014, whose sex class was not known.

|  | Peaceful | Agonistic | Play | Total |
| --- | --- | --- | --- | --- |
| BT | **479** | **73** | **211** | **763** |
| Males | 232 | 26 | 65 | 323 |
| Females | 245 | 47 | 144 | 436 |
| Unknown | 2 | 0 | 2 | 4 |
| OMBT | **92** | **130** | **244** | **466** |
| Males | 53 | 29 | 100 | 182 |
| Females | 39 | 100 | 136 | 275 |
| Unknown | 0 | 1 | 8 | 9 |
| Total | **571** | **203** | **445** | **1229** |
